# Reply to “Points of view in understanding trilobite eyes”

**DOI:** 10.1038/s41467-021-22228-7

**Published:** 2021-04-07

**Authors:** Gerhard Scholtz, Andreas Staude, Jason A. Dunlop

**Affiliations:** 1 Humboldt-Universität zu Berlin, Institut für Biologie/Vergleichende Zoologie, Philippstr. 13, 10115 Berlin, Germany; 2 Thermo Fisher Scientific, c/o Zuse Institut Berlin (ZIB), Takustr. 7, 14195 Berlin, Germany; 3 Museum für Naturkunde, Leibniz Institute for Evolution and Biodiversity Science, Invalidenstr. 43, 10115 Berlin, Germany

**Keywords:** Palaeontology

**Replying to** Schoenemann et al.* Nature Communications* 10.1038/s41467-021-22227-8 (2021)

In an article dealing with the fine structure of the compound eyes of two trilobite species we showed evidence for the existence of crystalline cones^1^. This finding was discussed in the context of the putative close relationship between Trilobita and Mandibulata. Schoenemann and Clarkson offered some critical comments on several aspects of our article^2^. Here, we rebut the points raised by these authors.

## The eyes of an undetermined asaphid

Schoenemann and Clarkson^2^ formulate a fundamental critique of our interpretation of the pattern of the eye of an undetermined asaphid. The authors raise three points of criticism. These relate to (1) a misunderstanding of the topology of the eye parts, (2) a wrong interpretation of the observed patterns, and (3) the neglect of the general structure of asaphid eyes.

Ad 1: Schoenemann and Clarkson^2^ claim that we did not understand what the correct positions of the facets were and that we interpreted the characteristic spikes of the eyes as crystalline cones (their Fig. 2b–d in ref. ^2^). However, it becomes obvious that this is not the case, when one reads the text on page 2 of our article and the caption of our Fig. 2 in ref. ^1^. The labels of the lenses in Fig. 2 in ref. ^1^ indicate that we placed the facets between two spikes and in the text (page 2) of our article^1^ and in Fig. 2 in ^1^ and its caption we state that the spikes are not the putative crystalline cones.

Ad 2: Schoenemann and Clarkson^2^ offer an alternative interpretation of the observed structures of the fossil compound eye. The general problem of fossils is that we are facing a pattern that is a mix of incomplete organismic structures and diagenetic impact. Hence, the pattern has to be interpreted to reconstruct the various processes that led to its current appearance. Furthermore, the interpretation of fossil structures is fundamentally based on comparisons with those of Recent organisms.

Therefore, it seems sensible to begin with the description of the observable eye pattern from our publication with a slightly different wording^1^. What we see from cross-and tangential sections (microscopic and μ-CT-based) is an outer calcite layer composed of a number of sub-layers. This layer forms hexagonal facets. Each of these facets shows a convex outer surface and in many cases a weakly convex inner surface. In cross-sections there are triangular pointed spikes underneath this facetted layer. These mark the interommatidial boundaries between the facets and they form regular rings beneath the facets as becomes evident from tangential sections underneath the eye’s surface. Like the layer of the facets, the spikes (rings) are calcitic but with an irregular internal structure and not layered as in the facets. These rings either circumscribe dark hollow spaces filled with matrix or white calcitic structures with a cone shape and a rounded inner end^2^.

**Fig. 1:**
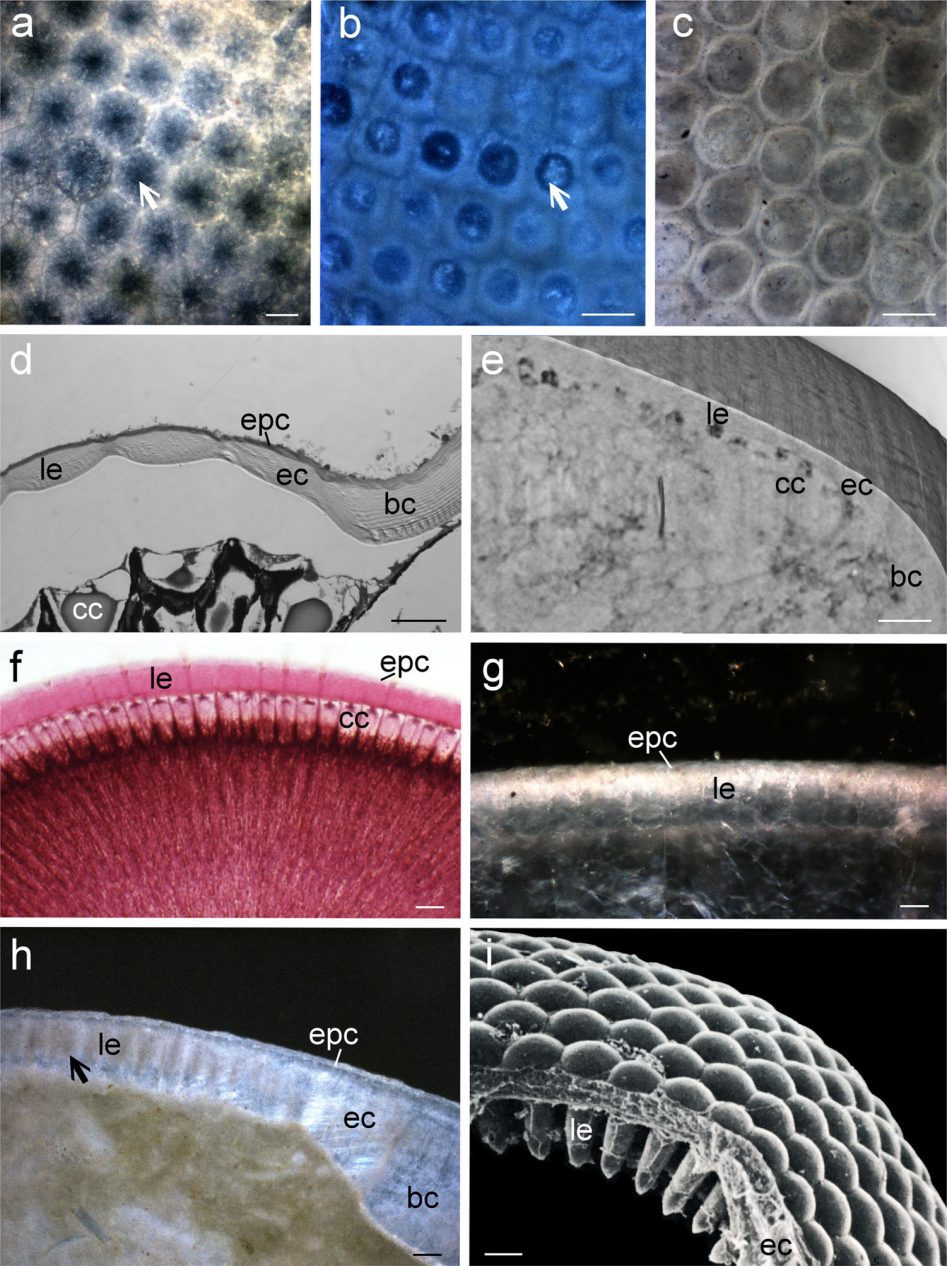
Comparison of various trilobite compound eyes and the eyes of extant crustaceans and hexapods. **a** Differential interference contrast light microscopical image (DIC) of a tangential section of the lenses of the Ordovician asaphid trilobite species *Symphysurus palpebrosus* (Naturhistoriska Riksmuseet, Stockholm: Ar0059423) with weathered lenses. Note the fuzzy appearance of the dark, degenerated parts (arrow). **b** DIC image of a tangential section of the lenses of the asaphid trilobite species *Asaphus expansus* (Naturhistoriska Riksmuseet, Stockholm: Ar0059396) with weathered lenses. Note the fuzzy appearance of the degenerated parts (arrow) Compare with **h**. **c** DIC image of a tangential section of the transition between lenses and the region of the putative crystalline cones (compare with **e**) of an undetermined asaphid (Naturhistoriska Riksmuseet, Stockholm: Ar0059402). Note the sharp circular margins and the overall different pattern, when compared with images **a** and **b**. **d** Transverse section (light microscopy) of the compound eye of the isopod *Paramphisopus palustris*. Due to shrinking processes during fixation the layer of the crystalline cones (cc) detached from the lenses (le). Note the confluent transition between the cuticle of the eye (ec) and the body (bc). The outermost layer is formed by the epicuticle (epc). (preparation: courtesy of Christian Wirkner, Rostock). **e** Transverse section (slice mode of a Synchroton scan) through the eye of an undetermined asaphid (Ar0019635) with the same confluent transition between the cuticle of the eye (ec) and the body (bc) as the isopod in **d** that differs from the pattern in asaphid species with columnar lenses (see **h**). cc, empty space of a putative crystalline cone; le, lens. **f** Transverse section (light microscopy) through the eye of a honey bee (*Apis mellifera*) (zoological collection Humboldt-Universität). Note the long columnar lenses (le) on top of the crystalline cones (cc). The epicuticle (epc) is also visible. **g** Transverse section (light microscopy) through the eye of the Ordovician asaphid trilobite *Nileus armadillo* showing a similar shape of lenses (le) to that of the bee (Naturhistoriska Riksmuseet, Stockholm: Ar 0059429). This lens shape does not preclude the existence of crystalline cones. Compare with the elongated lenses of *Asaphus expansus* (**h**) epc, epicuticle. **h** Transverse section (light microscopy) through the eye of the Ordovician asaphid trilobite *Asaphus expansus* (Naturhistoriska Riksmuseet, Stockholm: Ar0059396). This species has very elongated columnar cuticular lenses (le). Some of these are weathered and filled with mud (arrow) (compare with **b**). These long lenses are comparable with modern exocone eyes (see **i**) and may be a secondary achievement within asaphids. The pattern of the transition between the cuticle of the eye (ec) and the body (bc) differs from that of the undetermined asaphid shown in **e**. This indicates different lens types in the two species. epc epicuticle. **i** SEM image of a fracture of the exocone eye of an extant beetle (*Limnius perrisi*). Within the Coleoptera, the mandibulate crystalline cones (see **f**) are reduced and replaced by cuticular cones as part of the lenses (le). Ec eye cuticle. (image: courtesy of Hannes Paulus, Vienna). Scale bars 50 μm (**a**, **b**, **g**, **h**), 40 μm (**c**), 20 μm (**d**), 100 μm (**e**), 25 μm (**f**), 10 μm (**i**).

Schoenemann and Clarkson^2^ interpret these spikes/rings as the result of progressively degrading long hexagonal or columnar lenses. They state that these lenses are made of concentric layers of calcite with the weakest parts found in the center of the lenses. Hence, these inner parts degrade first leaving the outer layers as a ring around a hollow space. This hypothesis is interesting. However, Schoenemann and Clarkson only show a picture of the degrading inner tips of asaphid lenses, and the further process of lens degradation, as shown in a cartoon, is just an extrapolation of the observed pattern^1^. Furthermore, this hypothesis does not explain why these projections clearly form a perfect ring with very distinct margins underneath a hexagonal lens. This is particularly the case if the fuzzy margins, as shown in Fig. 1s,t^2^ of Schoenemann and Clarkson, are considered. In addition, the authors neglect the cone-like calcitic structure that is enclosed by some of the rings. If these cones were indications of complete lenses, one would not expect the existence of the rings. Finally, Schoenemann and Clarkson largely base their hypothesis of lens degeneration on specimens of the proetid species *Paladin eichwaldi shunnerensis* (see their Fig. 1r,t)^2^. However, the evidence is not compelling, since the images show the outer surface of the eye. Moreover, they do not mention that the specimen (Gr I 45668) has been artificially etched with EDTA to reveal the inner lens structures^3^. This questions the relevance of this example for their hypothesis. The argument of Schoenemann and Clarkson is further weakened by the analysis of transverse and tangential sections of the eyes of some asaphid species from the Lindström collection^4^ (Fig. 1). Some of these eyes show a degeneration of the lens structures (Fig. 1a, b, h). However, the patterns of the tangential sections are very different when compared with those of the asaphid described in our article (Fig. 1a–c). The hexagonal to squared shape of the lenses is visible even at a certain distance from the surface and the degeneration inside shows fuzzy margins.

**Fig. 2:**
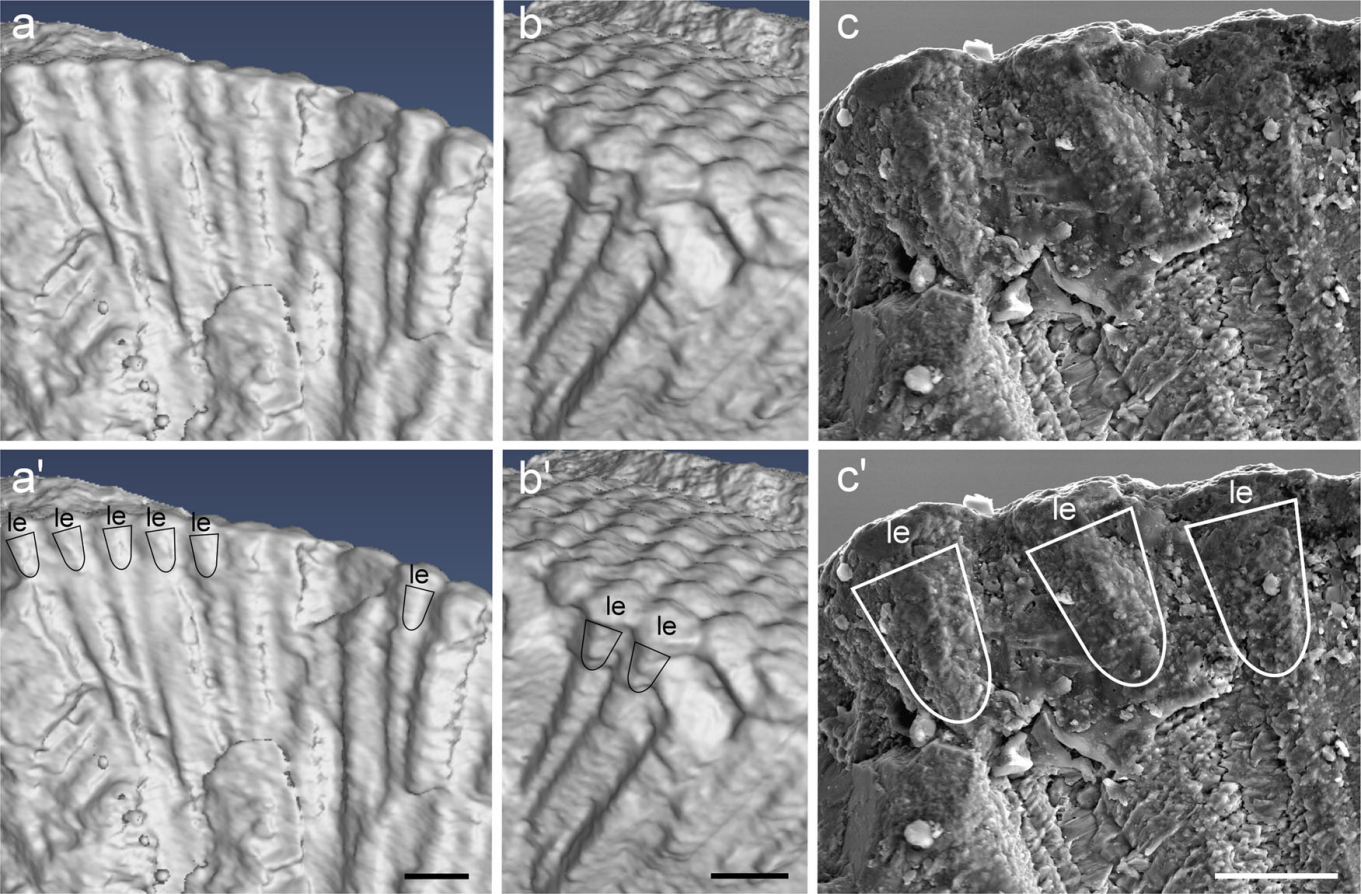
The compound eye of the Devonian trilobite *Archegonus warsteinensis* (Museum für Naturkunde, Berlin: MB.T 7303). The upper row (**a**–**c**) shows the compound eye structures using different techniques of visualization. **a**, **b** different perspectives using surface rendering of a μ-CT scan of different parts of the fractured eye. **c** An SEM picture of a part of image **a** slightly magnified. In all images, superficial lenses (le) (see **a**’–**c**’) are visible. Underneath them are cone-like structures that we interpret as fossilized crystalline cones. The cones are highlighted with black or white lines in the lower row (**a**’–**c**’). Scale bars: 100 μm (**a**’–**b**’), 50 μm (**c**’), they also refer to (**a**, **b**) and (**c**).

By contrast, our interpretation of the observed structures of the asaphid eye is as follows. The biconvex facetted layer represents the lenses. This view is supported by the fact that, as in modern arthropods, the cuticle of the eye lenses is confluent with the increasingly thicker cuticle of the head region (Fig. 1d, e, h). Like Schoenemann and Clarkson we suggest that the spikes underneath the lenses (we called them pointed projections, see our Fig. 2 in ref. ^1^) are diagenetically formed, but our interpretation is different. According to our view, they form in the interommatidial spaces in the area between the lenses and the top of the putative crystalline cones. The irregular internal structure of the spikes indicates that they are not part of the lenses. Hence, these pointed projections possibly circumscribe the upper part of crystalline cones. This view is based on the fact that some of the rings are filled with dark matrix and others with white round cone-like structures – the putative crystalline cones (Fig. 2c). These cones lie underneath the flat lenses and they are not confluent with the cuticle of the head. They are visible in the transverse section of the eye.

Schoenemann and Clarkson use the words epi- and exocuticle in a confusing way^2^. This is partly due to differences in terminology between trilobite and Recent arthropod eye parts. Whereas in Recent arthropods the term cornea is used for the cuticular lenses, trilobite researchers applied the word cornea just to the thin outer layer covering the lenses^5^. Yet, the cuticle of trilobites corresponds in its basic structure to that of Recent crustaceans including—following an external to internal sequence—an epicuticle, a layered exocuticle, and an endocuticle (principle layer)^6,7^. In addition, Schoenemann and Clarkson^2^ consider trilobite lenses as being of endocuticular origin. However, in all Recent arthropods, the cuticle of the eyes is formed by the same layers (but in different proportions) as the cuticle of the body^1,8,9^. Hence, all aspects of trilobite eyes speak in favor of an interpretation similar to that of Recent arthropod eyes. According to this view, the outer layer of trilobite eyes would be the epicuticle (Fig. 1). The cuticular lenses (even as calcite crystals) would be formed by or within all cuticular layers and would correspond to the cornea as in Recent arthropods.

Ad 3: Schoenemann and Clarkson^2^ state that several asaphid specimens show thick cylindrical or hexagonal lenses. They use this as an argument to dismiss our results that another asaphid species most likely formed crystalline cones. Of course, we were aware of the descriptions of these eyes by Lindström and Clarkson^4,10^ and we examined Lindström’s original preparations^1^. However, Lindström himself (p. 42)^4^ described this eye as “so peculiar that it seems worthy of being recorded”. Several aspects of the eye of this undetermined asaphid differ from those of other Asaphidae. The shape of the facets is largely hexagonal to round versus squared, rhomboid to hexagonal. The visual surface of the lenses is convex, whereas in all other asaphids the lenses were described as flat. The Asaphida is a very speciose and ecologically diverse group that existed from the Cambrian to the end of the Ordovician^11^. Hence, one can expect that asaphid eyes are not all of the same type. Examples for a putative diversity of asaphid eyes can be found in Lindström^4^, where he described eyes of *Nileus* and *Symphysurus* and in Bennett et al.^12^ who show the eye of an undetermined asaphid. All these species do not possess the long lenses of *Asaphus raniceps* and have different degrees of curvatures at the inner margin (see Fig. 1). Moreover, even the cylindrical lenses of some asaphids do not preclude the existence of crystalline cones as is demonstrated by hymenopterans, stomatopods, and some crabs (Fig. 1f, g)^13–15^. On the other hand, the elongated lenses of most asaphids resemble to a certain extent the exocone eyes of xiphosurans, some myriapods, and some hexapods (Fig. 1h, i). Hence, the situation in Asaphida seems comparable to that in beetles (Coleoptera). This highly diverse and speciose insect group evolved different types of compound eyes. The original condition was a characteristic mandibulate eye with a cuticular lens and a crystalline cone. By contrast, in several lineages of coleopterans exocone eyes evolved, in which the crystalline cone was reduced and replaced by a cuticular cone-like process similar to that of xiphosurans (Fig. 1i)^16^.

## The eyes of *Archegonus warsteinensis*

Schoenemann and Clarkson question our description of the parts of the ommatidia^2^. They dispute our findings concerning the rhabdom (1) and the crystalline cone (2), and they reinterpret one of our figures claiming that they detected a structure that is more likely to be a crystalline cone (3).

Ad 1: Schoenemann and Clarkson mention that the microvilli of the rhabdom are too small to be preserved^2^. However, we did not claim that individual microvilli are preserved. All that we said is that layered structure of the rhabdom might be visible. This is supported by the fact that the diameter of the putative rhabdom of *Archegonus warsteinensis* is in the range of modern crustaceans^1,17,18^.

Ad 2: As in the case of the asaphid species, Schoenemann and Clarkson misinterpreted our description of the eye of *Archegonus warsteinensis*. The structure marked by a blue arrow and the yellow bracket in their Fig. 1e^2^ (our Fig. 3f^1^) is not what we designated as a crystalline cone. By contrast, what we interpreted as part of the crystalline cone are the cone-like structures underneath the lenses as is indicated by the black line on top of the label “cc” in our Fig. 3f^1^. To connect a label and the described structure with a line is common practice in scientific illustrations. It is true that some of the crystalline cones of this image are not fully visible and somewhat distorted based on the perspective and some artifacts of the fracture. That these cones appear in the μ-ct somewhat smaller and shorter than in the SEM-picture of Fig. 3e^1^ is partly the result of the position in the fracture (a more peripheral versus a more central position within the lens and the crystalline cone) and a different perspective. In Fig. 3e^1^ we look at the internal eye structures from a ventro-lateral view, whereas in Fig. 3f^1^ from a dorso-lateral perspective (see also Fig. 2). Furthermore, Fig. 3f^1^ was not meant to demonstrate the shape of the crystalline cones but to indicate that these can be absent whereas the lenses remain. This allows the conclusion of a structural independence of cone and lens, which is not the case in exocone eyes^1^. We studied the eye of *Archegonus warsteinensis* with three different techniques, namely light microscopy, SEM, and μ-ct. Only structures that were congruent in all methods were used for our interpretation. To clarify the issue, we present additional images here to show what part we interpret as the crystalline cone (Fig. 2).

Ad 3: Schoenemann and Clarkson pick one of our images in which they claim to see a more likely example of a crystalline cone (Fig. 2f, g)^2^. This interpretation is based on a comparison with crystalline cones found in the compound eyes of a Jurassic crustacean. However, this structure appears in only one ommatidium of *Archegonus* and a closer look reveals that it is much too long to be a crystalline cone. Hence, we conclude this it is an artifact and thus we reject the claim of Schoenemann and Clarkson^2^.

In summary, we think that the criticism and the alternative interpretation of Schoenemann and Clarkson are not compelling^2^. There is still enough evidence to adhere to our interpretation of the structures in the compound eyes of both trilobite species, studied by us^2^. In the light of the new publication by Schoenemann and Clarkson^19^, in which they add new evidence for the existence of crystalline cones in Trilobita, one can conclude even more strongly that the trilobite stem species possessed compound eyes with crystalline cones similar to modern day myriapods, crustaceans, and hexapods^1^.

### Reporting summary

Further information on research design is available in the Nature Research Reporting Summary linked to this article.

## Supplementary information

Reporting Summary

## Data Availability

We used the original trilobite material from Gustaf Lindström housed in the Naturhistoriska Riksmuseet, Sektionen för Paleozoologi, Stockholm (Sweden). The material comprises a number of microscopic preparations of the eyes of various trilobite species among them two of an undetermined asaphid specimen from the Ordovician (Gotska sandön, Gotland, Sweden) (Ar0059402). From this specimen, the anterior part exists from which Lindström had cut off parts for microscopic preparations (Ar0019635). Other specimens from the Lindström collection used in this study: *Symphysurus palpebrosus* (Ar0059423, *Asaphus expansus* (Ar0059396), *Nileus armadillo* (Ar 0059429). Specimens of *Archegonus* (*Waribole*) *warsteinensis* from the upper Devonian (Fammenian) of Germany (Kalvarienberg/Kallenhardt) were collected by Dieter Korn. This material is housed in the Museum für Naturkunde, Berlin, Germany (MB.T 7303). The slide of the bee eye is housed in the zoological collection of the Humboldt-Universität zu Berlin. The datasets generated during and/or analyzed during the current study are available in the Humboldt-Universität zu Berlin: edoc-server repository, 10.18452/20002.
